# Characterization of Microstructure, Chemical, and Physical Properties of Delignified and Densified Poplar Wood

**DOI:** 10.3390/ma14195709

**Published:** 2021-09-30

**Authors:** Jiajun Wang, Junliang Liu, Jianzhang Li, J. Y. Zhu

**Affiliations:** 1 Research Institute of Wood Industry, Chinese Academy of Forestry, Beijing 100091, China; wangjiajun@caf.ac.cn (J.W.); liujunliang@caf.ac.cn (J.L.); 2 College of Materials Science and Technology, Beijing Forestry University, Beijing 100083, China; 3 USDA Forest Products Lab, Madison, WI 53726, USA

**Keywords:** delignification, densification, microstructure, chemistry, dimensional stability

## Abstract

Wood is an attractive and inherently sustainable alternative to many conventional materials. Recent research on improving wood mechanical strength emphasizes wood densification through the partial removal of lignin and hemicelluloses, therefore the chemical and physical properties of delignified and densified wood require further investigation. In this study, poplar wood samples were subjected to alkali and maleic acid hydrotropic delignification with varying degrees of lignin and hemicellulose removal followed by hot pressing, and the microstructure, chemical properties, and dimensional stability of densified wood through delignification were evaluated. The results showed that the complete wood cell collapse was observed near the surface of all the delignified wood blocks, as well as some micro-cracks in the cell walls. The chemical analysis indicated that delignification occurred mainly near the surface of the wood blocks and enhanced hydrogen bonding among the aligned cellulose fibers. For dimensional stability, the set recovery decreased with the increase in alkali dosage, and the considerable fixation of compressive deformation was obtained by a post-densification hydrothermal treatment at 180 °C. These results have demonstrated that the densified wood with delignification can be easily fabricated using the proposed method, and the densified wood exhibited great potential to be used as a sustainable material.

## 1. Introduction

Wood is a natural, renewable, and abundant structural material, which has been widely used in residential construction, bridges, and furniture [1]. Increasing wood utilization through conservation not only can reduce the carbon footprint but promote sustainable and healthy forest management. Wood treatments through the thermal process [2] and resin impregnation [3] have been common practices to improve the dimensional stability and durability of wood; nevertheless, considerable disadvantages remain, including harmful reagents and difficulties at the end of life [3,4,5]. Specifically, the densification through hydro-thermo treatment has also been applied to improve wood mechanical strength, especially for woods with low densities, such as fast-growing and small-diameter trees in overpopulated forests. Wood after delignification treatment can be compressed under low pressures, and the densified wood exhibited improved strength, better than with thermal treatment. Recent studies of wood densification through delignification have demonstrated great potential for those applications that require high strength performance [6,7].

Wood densification through pressing that collapses the wood lumina and the porous wood cell walls results in improved mechanical properties. With delignification, wood densification can be achieved at substantially lower pressures facilitated by hydrogen bonding, a significant advantage for reducing both the processing cost and the defective products caused by high-pressure-induced cracking [8,9]. It will be interesting study the chemical and physical properties of delignified and densified wood. Due to the anisotropy of wood, the delignification of bulk wood is inherently inhomogeneous with inconsistent chemical changes. Therefore, it is desirable to monitor the lignin distribution in the cell wall and increase the knowledge of the local property changes in the delignified and densified wood. Since lignin fluoresces over a broad range of wavelengths, the wood cell walls are naturally fluorescent due to the presence of lignin, whose location may be identified by confocal laser scanning microscopy (CLSM) [10]. Moreover, dimensional stability is critically important for most applications in the context of materials. Densification through hot pressing leads to plastic deformation of the wood cells and bulk wood [11]. This process is partially reversible, i.e., the thickness of densified wood can increase in contact with moisture [12]. This behavior, known as “set recovery” or “spring-back effect” [13], compromises the mechanical strength of densified wood. To stabilize the dimension, the current pathways for wood deformation fixation include resin impregnation [14], cross-linking reaction [15], heat treatment [16], etc. Heat treatment has been employed at various temperatures (160–220 °C) and media reactions, such as vacuum, nitrogen, steam, and oil [17]. High temperature hydrothermally treated wood exhibits enhanced anti-weathering resistance and improved dimensional stability due to the release of the wood’s internal stresses [16] by means of micro-cracks in the cell wall, the degradation of hemicelluloses, the loss of C=O linkages to the aromatic skeleton in lignin, and the increase in crystallinity [18]. However, it is unclear if this set recovery is approximately more or less pronounced for the densified wood through delignification occurring with hemicellulose dissolution.

Our previous study on wood densification through delignification focused on the improvement of mechanical strength, with little attention paid to microstructure, lignin distribution, or the fixation of compressive deformations, which hinders its potential applications in improving the mechanical properties of low-density wood [19].

The objective of the present study is to investigate the microstructural, chemical, and physical properties of delignified and densified wood for sustainable application.

## 2. Material and Methods

### 2.1. Materials

**Figure 1:**
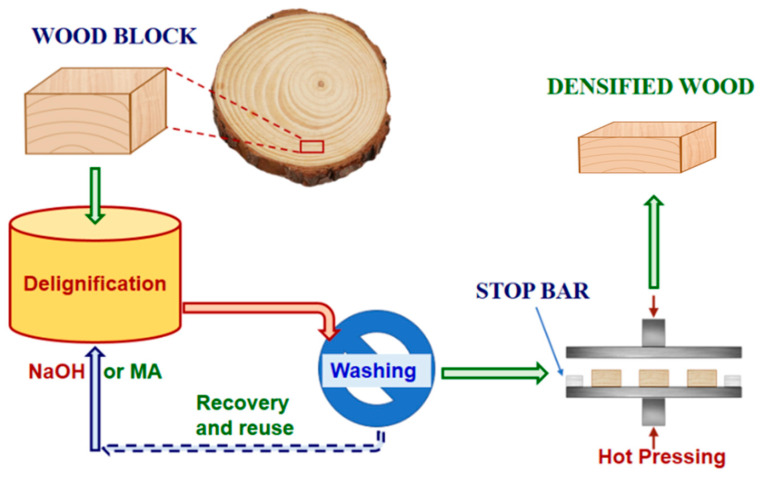
A schematic flow diagram showing wood block cutting, chemical delignification, and densification through hot-pressing.

Poplar tree (*Populus deltoides* Bartr. ex Marsh × *P. nigra* L.) was harvested in December 2018 from Hugo Sauer Nursery, USDA Forest Service, Northern Research Station, Rhinelander, WI, USA. The wood block samples, with the dimensions of 100 mm (longitudinal) × 20 mm (tangential) × 10 mm (radial), were cut from fresh wood logs (Figure 1). Wood materials within a radius of 10 mm from the center of each wood log were discarded to reduce the effects of juvenile wood on the wood block properties.

Maleic acid (MA) and sodium hydroxide (NaOH, 98%) were the ACS reagent grade and purchased from Sigma-Aldrich (Burlington, MA, USA).

### 2.2. Delignification

Aqueous NaOH solutions at 0, 1, 2, and 6 wt% concentrations and an aqueous MA solution at 50 wt% were prepared by dissolving appropriate amounts of NaOH or MA in water. Alkaline wood delignification was conducted at 155 °C for 30 min using NaOH solutions at a liquor-to-wood ratio of L/W = 3:1 in a 1 L bomb reactor. Three 1 L bomb reactors were placed in a 21 L rotating wood pulping digester heated by a steam jacket, as previously described [20]. These delignification runs were denoted as *An*, with *n* = 0, 1, 2, 6 representing NaOH concentrations in wt%. A0 without NaOH is a pure hydrothermal treatment. Acid hydrotropic delignification was conducted using MA hydrotropic fractionation [21] at 100 °C for 30 min in a flask submerged in a glycerol heating bath. The MA treatment run is labeled as *M50*. After delignification, all the wood blocks were washed several times using tap water to a pH of 7 ± 0.2.

### 2.3. Wood Densification

After washing the wood blocks, they were densified in the wood radial (thickness) direction. The samples were compressed from an initial thickness of 10 mm to a final thickness of approximately 8 mm at a pressure of 1.0 MPa for 15 min at 150 °C by using two stop bars of a thickness of 8 mm. One set of wood samples, labeled as A2N and treated with NaOH of 2 wt%, was pressed without stop bars but under the same densification conditions used to achieve maximal densification. The preparation conditions, densities, and chemical compositions of the untreated and densified wood samples were cited from our previous study (Table 1) [19]. After densification, the wood samples were dried in a climate chamber at 20 °C for two weeks at a relative humidity of 65%.

### 2.4. Post-Densification Hydrothermal Treatment

The heat treatments of the untreated and densified wood samples were carried out at 180 °C for 2 h in an airtight vessel of 0.13 m^3^ in volume with no more than 2% oxygen content. The vessel was heated by injecting saturated steam as described by Gao et al. [22]. After the treatment, the samples were cooled, dried, and weighed. The post-treatment run was labeled as Untreated_p_, M50_p_ A2N_p,_ and An_p_ with *n* denotes the NaOH concentration in the alkali treatment.

### 2.5. Analysis of Microstructure, Chemical Composition and Physical Properties

For the scanning electron microscopy (SEM) analysis, densified and untreated poplar wood samples were coated with a 6-nm thick layer of gold using a sputter coater after fixing, cleaning, and drying. Finally, the sections were examined on a GeminiSEM 300 (Zeiss, Germany) under a vacuum.

The Fourier transform infrared (FTIR) spectroscopic analysis of untreated and densified wood treated at NaOH = 2 wt% was conducted using a spectrometer (Spotlight 400, PerkinElmer, Waltham, MA, USA). Before the analysis, the samples were ground and mixed with potassium bromide (KBr). All spectra were collected in the wavenumber range between 3000 and 400 cm^−1^, with a resolution of 4 cm^−1^ and at least 32 scans of each sample. Three duplicates were scanned for each treatment, and the spectra were treated by baseline correction and normalization.

For the CLSM autofluorescence analysis, densified wood A2 and the untreated wood sample were first soaked in deionized water. Sections of 10 μm thick were cut from the two wood samples using a microtome (Leica RM2015, Weztlar, Germany) and subsequently mounted on slides. The microtome-cut samples were then directly examined for autofluorescence with a CLSM (Leica DM IRB2, Weztlar, Germany) using 405 nm as the excitation wavelength. The exposure was kept strictly identical so to ensure comparability among the samples.

Water absorption (WA), thickness swelling (TS), and set recovery (SR) were measured within an area of 20 mm × 20 mm of a densified wood sample cut from the middle area of the densified wood of 100 mm × 20 mm. The thickness and weights of all the untreated and densified wood samples were then measured. The samples were soaked in water for 16 days and then oven-dried. The weight and dimensions of a sample under saturation and oven-dried conditions were recorded. The WA was evaluated as

(1) $$\mathrm{WA}=\frac{m-m_{0}}{m_{0}}\times 100\%$$

where *m* is the wet weight after immersion in water for 16 days, and *m_0_* is the oven-dried weight before immersion in the water.

TS was determined by measuring the thickness of the samples using

(2) $$\mathrm{TS}=\frac{t_{max}-t_{0}}{t_{0}}\times 100\%$$

where *t_max_* is the thickness after water saturation and *t*_0_ is the thickness before immersion in the water.

SR was calculated as

(3) $$\mathrm{SR}=\frac{t_{r}-t_{0}}{t_{u}-t_{0}}\times 100\%$$

where *t_r_* is the thickness of soaking the saturated densified wood block after oven drying, *t_u_* is the thickness of the corresponding undelignified and undensified raw wood blocks, and *t*_0_ is thickness before immersion in the water.

Thermogravimetric analysis of all the wood samples was carried out by a thermal analyzer (STA449F3, Netzsch, Germany). Milled wood samples were scanned from 25 °C to 700 °C at a heating rate of 10 °C/min in a nitrogen flow of 40 mL/min.

## 3. Results and Discussion

### 3.1. Microstructure of Densified Wood

**Figure 2:**
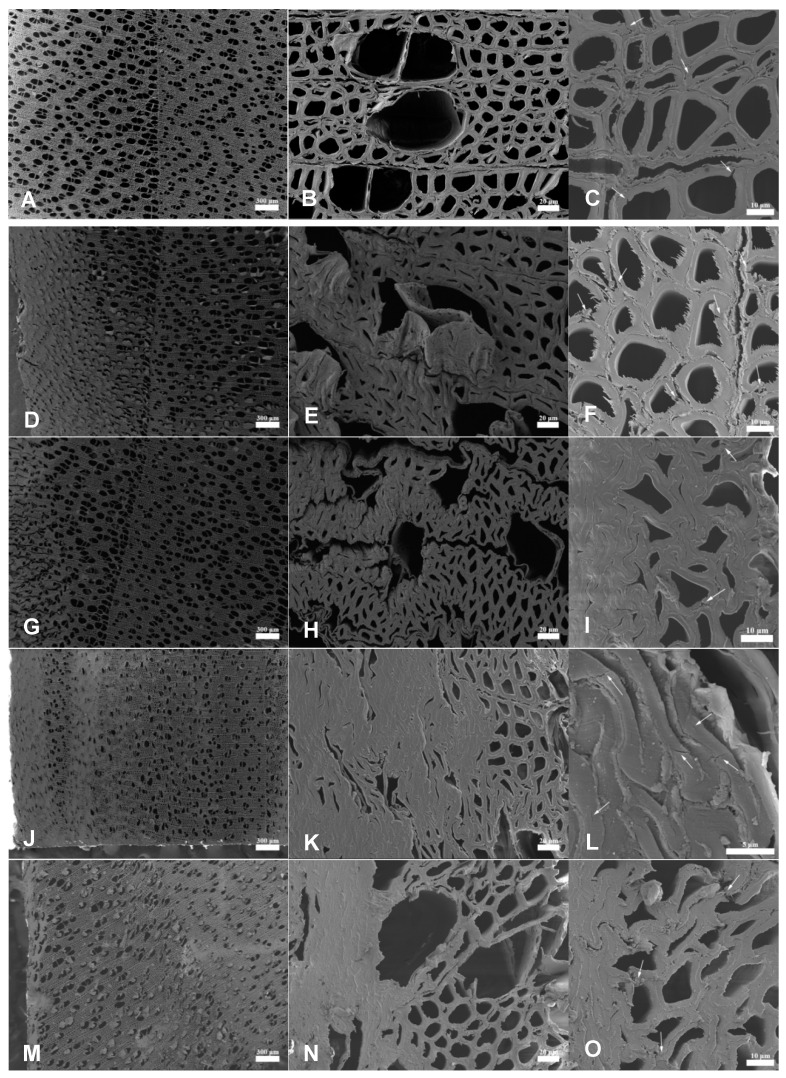
The SEM images showing wood cell microstructures: (**A**–**C**) untreated wood; (**D**–**F**) *A0*; (**G**–**I**) *A2*; (**J**–**L**) *A2N*; and (**M**–**O**) *M50*. Left column: scale bars = 300 µm; middle column: scale bars = 20 µm; right column: scale bars = 10 µm, except L = 5 µm.

The microstructures of the untreated and densified wood samples were characterized by SEM. Figure 2A–C shows the microstructure of the untreated poplar wood at three magnifications. With these dimensions in mind, it may be said that densification through compression changed the microstructure of densified wood cells, such as buckling the cell walls and decreasing the volume of the void spaces or porosity, as shown in the left column of Figure 2, but substantial cell collapsing mainly occurred near the surface of the wood blocks due to the low pressure of 1 MPa loaded. When pressing without the stop bars (Figure 2J), cell collapsing was also observed outside the surface region towards the center of the wood, with weak mechanical strength either due to defects or chemical delignification. Delignification in a high-temperature aqueous system loosened wood cell structure and reduced the transverse rigidity of the cell walls, which facilitated densification that resulted from cell collapse and cell deformation.

The hydrothermal treatment, which does not delignify wood, resulted in a small decrease in the thickness in A0. No complete cell collapse was observed from A0 (Figure 2D,E), even near the surface of the wood block; nevertheless, cell deformation that decreased wood lumina size throughout the wood thickness direction was observed (comparing with natural poplar wood as shown in the first row of Figure 2), in agreement with those observed from the thermal densification treatment of pine wood [23]. In contrast, complete cell collapse was observed near the surface of all the chemically treated wood blocks (Figure 2G,J,M), as previously reported in optical microscopy [19]. This phenomenon indicated that delignification occurred in the wood layers near the wood block’s external surfaces due to the limited chemical penetration depth, especially for the MA-treated sample (Figure 2M) with the requirement of a minimal hydrotropic concentration for effective delignification [21,24]. Other studies have also reported that the extent of the cell collapse had a significant effect on the mechanical and physical properties of the densified material [13,25]. Additionally, the increasing wood surface density was effective in preserving wood bulk (thickness) so to reduce energy consumption in pressing and to meet application specifications.

Increasing the magnification in SEM imaging provides a better-localized view of the wood cells, specifically to examine the cracks within cell walls, as shown in the right column of Figure 2. The cracks were clearly visible from the undensified and untreated poplar samples, as marked by the arrows (Figure 2C) and in the cross-section of the densified wood samples (the other images in the right column of Figure 2), which was consistent with those studies reported previously [26]. This result suggested that the observed cracks in the densified wood may not be induced by densification; rather, they might have resulted from the drying process during the sample preparation [27]. A2N was pressed without stop bars, and more cell collapse and deformation were observed, as discussed previously. It also exhibited some micro-cracks on the cell wall that may have been caused by compression force [28]. These cracks provided cellulose fibers with the physical space for relaxation to affect the set recovery [29,30].

### 3.2. Chemical Properties of Densified Wood

**Figure 3:**
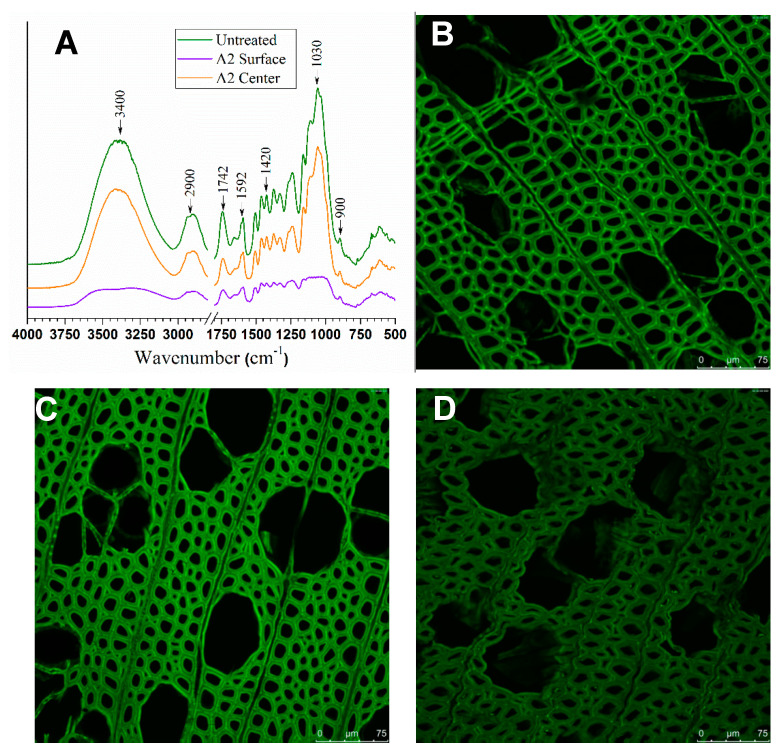
(**A**): The FTIR spectra of an untreated wood sample, and wood sections from surface and center layers of *A2*; (**B**–**D**): The fluorescence image (with an excitation wavelength at 405 nm) of lignin in an untreated wood sample; (**B**) the center layer (**C**) and surface layer (**D**) of alkali-treated *A2.* Scale bars = 75 µm.

Delignification changes the wood chemical composition, which affects wood cell collapse during wood densification. The non-uniformity in delignification resulted in the observed surface densification phenomenon even when pressing was applied without the stop bars, as discussed earlier. Chemical analyses of microtome wood sections from the different depths of the wood block thickness directions may reveal chemical compositional changes. The FTIR spectra of the untreated wood and the samples from the surface and center layers of A2 are shown in Figure 3A. No significant differences were observed from the spectra between the untreated poplar wood and the sample from the center of the alkali-treated wood A2. Both samples showed strong peaks at 3400 cm^−1^ and 1030 cm^−1^, in contrast to the sample from the surface of the A2 wood that exhibited substantially weakened peaks at these two wavenumbers. These two peaks were attributed to the O–H stretching of cellulose [31] and the cellulose backbone, respectively, indicating cellulose consolidation by alkali at elevated temperatures. The decreased intensity at 1742 cm^−1^, attributed to C=O stretching in hemicelluloses, lignin, and extractives, in the spectra of the two samples from alkali-treated wood suggested the removal of chemical components, and this was especially notable for the sample from the wood surface with a greater decrease in signal peak intensity. Lignin removal was evident from the peaks at 1505 cm^−1^, 1592 cm^−1^, and 1648 cm^−1^ and corresponded to aromatic skeletal vibration in lignin [32,33,34]. The intensities of these peaks were substantially decreased for the alkali-treated sample from the wood surface.

The CLSM provides complementary information about lignin distribution. Fluorescence images of lignin in the untreated wood and wood sections from the surface and center layers of A2 were obtained under the same excitation laser wavelength at 405 nm. The images of the untreated wood (Figure 3B) and the center layer of alkali-treated wood (Figure 3C) showed similar fluorescence reactions (i.e., bright green as shown in wood cell corners, middle lamella, and secondary wall), suggesting similar lignin concentrations between them. However, the image of the sample from the surface layer of the alkali-treated wood sample A2 (Figure 3D) showed a much less intense fluorescence than that observed from the untreated wood and the sample from the center of the wood block in Figure 3B,C. This result indicated that more lignin was removed from the sample of the alkali-treated wood A2 surface. Furthermore, the middle lamella of the sample from the A2 surface layer exhibited a fluorescence signal intensity relatively stronger than that of the secondary cell wall of the same sample when compared with the untreated poplar sample or the sample from the A2 center layer, suggesting that lignin was preferentially removed from the secondary cell walls by alkaline treatment.

### 3.3. Physical Properties of Densified Wood

#### 3.3.1. Dimensional Stability

**Figure 4:**
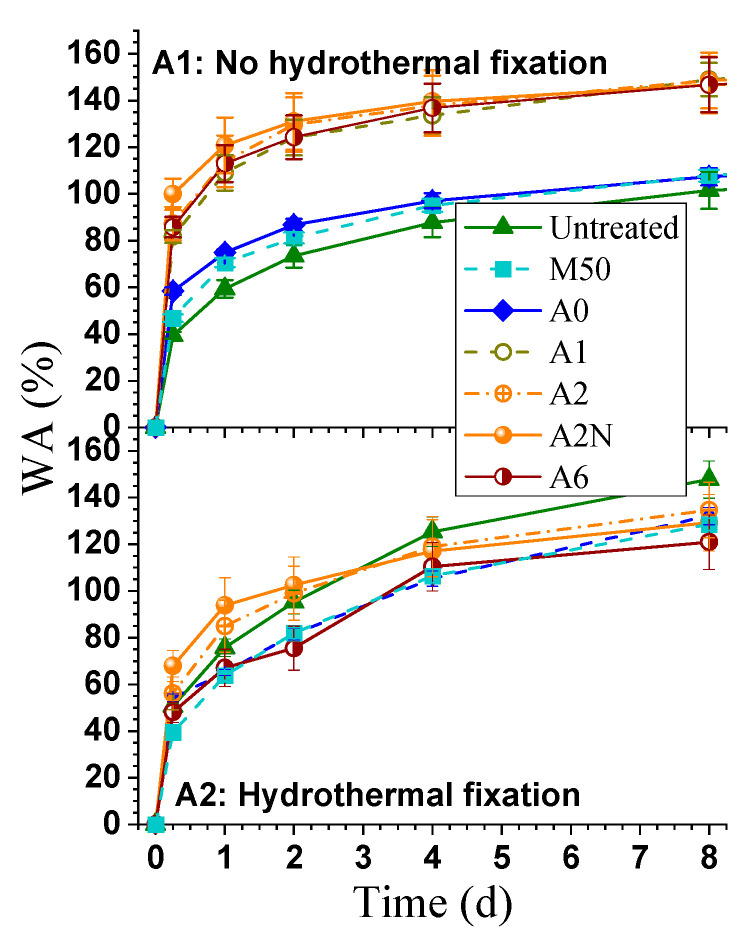

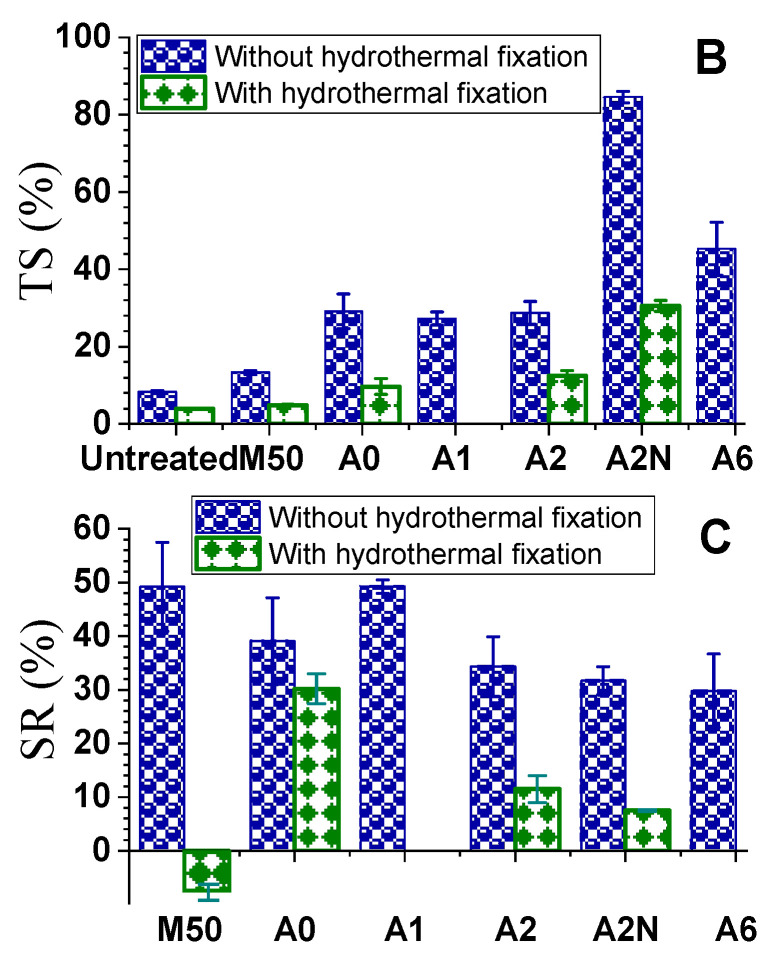
The effect of hydrothermal fixation on the dimensional stability of delignified and densified wood samples: (**A1**,**A2**) water absorption (WA) without (**A1**) and with (**A2**) hydrothermal fixation; (**B**) thickness swelling (TS); and (**C**) set recovery (SR).

Water absorption (WA), thickness swelling (TS), and set recovery (SR) were tested to evaluate wood dimensional stability. The WA of all wood samples initially increased rapidly and then gradually reached their final value with an extended soaking time (Figure 4A). The WAs of all samples on the first day accounted for more than 50% of the total WA in 16 days. Furthermore, the four wood samples from the alkali treatment had a much greater WA than those three with minimal or without lignin dissolution, as more hydrophilic sites of the cellulose were exposed after delignification. However, the effects of the extent of lignin removal on WA among the three alkali-treated wood samples were not visible, mainly due to the opposite effects of the increasing hemicellulose dissolution that decreased WA at a greater delignification with a higher alkali dosage, as well as the attainment of the maximum absorption capacity. The terminal WA values were approximately 150% for the alkali-treated wood samples, similar to those of unbleached wood fibers as reported in the literature [35]. Post-densification hydrothermal fixation reduced the WA of all alkali delignified samples and decreased the variation in WA between the set from alkali delignification and the rest of the samples (Figure 4A).

The TS of the densified wood samples increased with the amount of mass loss through the thermo-chemical treatment and with the density of the densified wood (Figure 4B). The sample without the stop bars in pressing, A2N, exhibited a much higher TS (over 100%) than with the stop bars, A2, as the internal stresses introduced during hot-pressing were more easily relieved when the compression ratio was lower [6,19]. Moreover, from the definition of TS, A2N with a much less initial thickness results in a large TS value. TS of A2N_p_ decreased sharply to 30.5% from 84.6% post-treatment.

The average set recoveries (SRs) for the densified wood samples are shown in Figure 4C. The elastic strain energy stored in semi-crystalline micro-fibrils and the lignin of the wood is the principal reason for the compression set recovery [12,36]. It appeared that increasing delignification or component removal slightly reduced the SR (Figure 4C). *M50_,_* which had the least amount of delignification or mass loss (Table 1), showed the highest SR of approximately 50%, while *A6*, with the most delignification and mass loss, had the lowest SR of 30%, indicating that delignification improved the hydrogen bonding of the cellulosic materials in wood to result in a decreased SR. The dissolution of lignin and hemicelluloses also created the physical spacing for cellulose relaxation to reduce SR [12]. Hydrothermal fixation also significantly reduced set recovery SR, as shown in Figure 4C. The hydrothermal fixation treatment broke down some hemicellulose cross-links responsible for the memory effect in wood, coupled with softening remaining lignin, and perhaps the formation of covalent bonds in the deformed position during the hydrothermal treatment [37]. The increasing delignification by increasing alkali dosage, and decreased SR, facilitated the removal of hemicellulose cross-linkage (Table 1) and the formation of hydrogen bonds between the exposed cellulose nanofibers during the dehydration after water swelling [38]. The decreases in SR for A2N_p_ and A6_p_ were much more significant, which decreased to 31.7% from 7.5%, and 29.8% from 3.8%, respectively (Figure 4C). Similarly, Xiang et al. [18] showed that the superheated steam contributed to reducing the SR from 47.1% to 23.9% for surface densified wood. Additionally, M50_p_ had a shrinkage rate of 7.3%, which could be attributed to the dissolution of hemicelluloses by the hydrolysis of the remaining acid under high temperatures.

#### 3.3.2. Thermal Stability

**Figure 5:**
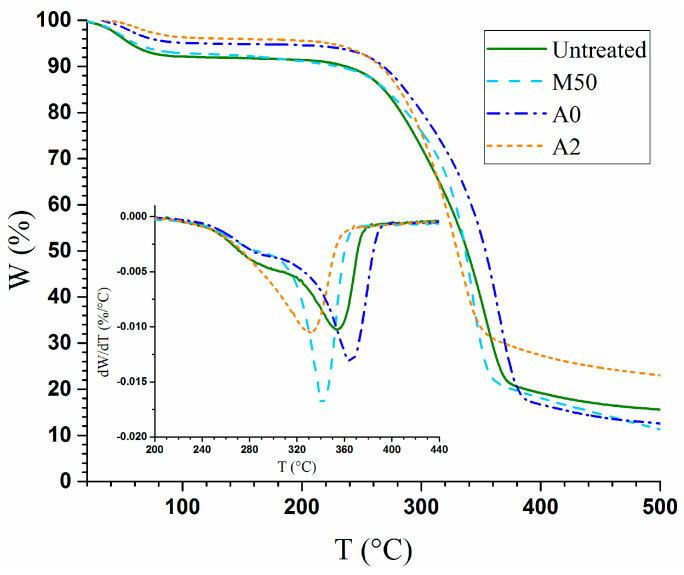
The thermal gravimetric analysis of untreated poplar and densified poplar wood samples.

The thermogravimetric (TG) analysis is one of the most common techniques used to analyze the thermal stability of woody materials. The TG and its derivative (DTG) curves of the untreated and densified wood samples are shown in Figure 5. The thermal degradation of the samples consists of three stages. The first stage took place in the range from 25 °C to 150 °C with a weight loss of approximately 8.2% for untreated wood samples, whereby the weight loss can be attributed to dehydration and the loss of low molecular weight volatiles. The high-temperature hydrothermal and alkali treatments at 150 °C apparently eliminated some low-molecular-weight compounds, resulting in a lower mass loss in this first stage than that from the untreated wood or the low-temperature MA treatment. The weight loss for A2 at the end of the first stage was 4.0%, which was the lowest of all samples (Figure 5), because alkali treatment also removes extractives. The majority of wood mass loss occurred in the second stage, from 150 °C to 400 °C, due to the thermal degradation of hemicelluloses, cellulose, and lignin [19]. This stage had a mass loss of approximately 85% for the untreated wood samples, with a temperature corresponding to the maximum mass loss, T_m_ = 354 °C (see insert in Figure 5). Hydrothermal treatment increased T_m_ to 366 °C, the highest of all the samples tested, due to its high lignin content of 25% and enriched by the dissolution of hemicelluloses (Table 1) that has a much lower degradation temperature than lignin [39,40]. The lower T_m_ for A2 and *M50* than the T_m_ of untreated wood was likely caused by cellulose depolymerization in addition to delignification. The decomposition of hemicelluloses and disordered cellulose usually has a characteristic shoulder in the curve of the weight loss derivative (dW/dT) [41]. However, this characteristic shoulder was not present for the alkali-treated and densified sample A2 due to the high level of hemicellulose dissolution during alkali treatment (Table 1) and the consolidation of disordered cellulose by the high-temperature alkali treatment. The decreased degradative temperature of the samples was attributed to delignification, which is consistent with the previous result [42].

## 4. Conclusions

The microstructural, chemical, and physical properties of wood after partial delignification and densification processes were evaluated in this study. Cell collapsing took place mainly near the wood surface and in the regions with low mechanical strength, with deformation occurring in the entire wood depth direction. Chemical analyses indicated that delignification mainly occurred in the wood surface layer, with the center layer remaining chemically unchanged. Increasing lignin removal reduced the dimensional stability of the densified wood (without thermal treatment), and the post-densification hydrothermal fixation treatment considerably improved the dimensional stability with low mass loss. These results demonstrated that wood densification through delignification could be a viable avenue for improving wood properties with broad applications.

## Figures and Tables

**Table 1:** List of wood delignification and densification conditions, and chemical composition [19].

Sample	Delignification, Densification			Chemical Composition					
	T (°C)	Time (min)	Stop bar	Density (g/cm^3^)	Solids Yield (%)	K Lignin (%)	Glucan (%)	Xylan (%)	Mannan (%)
Untreated	—	—	—	0.463	100	23.6 (100)	45.2 (100)	18.4 (100)	3.2 (100)
*A0*	155	30	Yes	0.554	95.2	25.9 (101)	47.0 (99)	17.9 (92)	3.3 (91)
*A1*	155	30	Yes	0.511	90.4	22.3 (86)	51.5 (103)	18.8 (92)	2.7 (85)
*A2*	155	30	Yes	0.536	86.7	24.6 (90)	48.7 (94)	17.9 (84)	2.3 (79)
*A2N*	155	30	No	0.811	—	—	—	—	—
*A6*	155	30	Yes	0.569	74.3	19.9 (63)	56.1 (92)	16.2 (65)	1.3 (57)
*M50*	100	60	Yes	0.507	98.0	24.5 (102)	50.5 (110)	18.2 (97)	3.6 (96)

Note: the numbers in the parentheses are component yields.

## Data Availability

Not applicable.
